# Classification of the risk of transmission of vaccine-preventable diseases among children under 2 years of age in the municipality of São Paulo, Brazil

**DOI:** 10.1590/1980-549720260035

**Published:** 2026-08-07

**Authors:** Mariana de Souza Araújo, José Elisomar Silva de Santana, Melissa Palmieri, Luciana Ursini Nunes, Thaís Moreira Oliveira, Maria Eduarda Viana Oliveira, Guilherme Augusto Veloso, Thales Philipe Rodrigues da Silva, Fernanda Penido Matozinhos

**Affiliations:** I Prefeitura Municipal de São Paulo, Municipal Health Department, Health Surveillance Coordination Office - São Paulo (SP), Brazil.; II Prefeitura Municipal de São Paulo, Municipal Health Department, Health Surveillance Coordination Office, Municipal Immunization Program - São Paulo (SP), Brazil.; III Universidade Federal de Minas Gerais, School of Nursing - Belo Horizonte (MG), Brazil.; IV Universidade Federal Fluminense, Institute of Mathematics and Statistics, Department of Statistics - Niterói (RJ), Brazil.

**Keywords:** Vaccination coverage, Risk management, Vaccination, Public health surveillance

## Abstract

**Objective:**

To analyze the classification of the risk of transmission of vaccine-preventable diseases among children under 2 years of age in a large Brazilian municipality in 2022 and 2023.

**Method:**

Ecological epidemiological study using secondary data on vaccination coverage (VC) for nine immunobiologicals recommended in the vaccination schedule for children under 2 years of age. Immunobiological dose data were obtained from the National Immunization Program Information System (*Sistema de Informações do Programa Nacional de Imunizações* - SI-PNI), while data were collected from the Ministry of Health website and filtered by place of residence. Data were analyzed using the R software package.

**Results:**

In 2022, 100% of the Health Surveillance Units (*Unidades de Vigilância em Saúde* - UVIS) showed low VC for the second dose of the measles-mumps-rubella (MMR) vaccine. In the same year, 61% and 54% of the UVIS achieved adequate VC for the hepatitis A and human rotavirus vaccines, respectively. In 2023, improvements in VC were observed for most vaccines, except for the meningococcal C and varicella vaccines, which showed a reduction in the number of UVIS achieving adequate VC. Regarding the classification of the risk of reintroduction of vaccine-preventable diseases, the proportion classified as “low and very low” increased from 0% in 2022 to 10.7% in 2023.

**Conclusion:**

VC improved in 2023 for most vaccines analyzed across the UVIS. However, variations in VC were observed according to vaccine type and year. These findings reinforce the need to maintain regional actions aimed at achieving vaccination targets, given the indications of a recovery in VC.

## INTRODUCTION

Established in 1973, Brazil’s National Immunization Program (*Programa Nacional de Imunizações* - PNI) is considered one of the country’s most significant public health achievements^1^. The program provides high-quality vaccines to populations throughout the national territory and has contributed substantially to improving public health outcomes in Brazil^2^.

Despite the undeniable success of PNI over the years, a progressive and concerning decline in vaccination coverage has been observed globally and in Brazil since 2016^1^, a trend that was further exacerbated by the COVID-19 pandemic^3^. According to data from the Brazilian Ministry of Health (MoH), vaccination coverage (VC), which reached 97% in 2015, declined to 75% in 2020^4^. Furthermore, according to the World Health Organization (WHO), nearly 26% of the pediatric population in Brazil had not received any vaccine doses in 2021^4^.

The decline in VC results from multiple factors^1^, including vaccine hesitancy^5^, defined as the delay in acceptance or refusal of vaccination despite the availability of vaccination services^6^. In 2019, the WHO identified “vaccine hesitancy” as one of the ten greatest threats to global health^6^. Vaccine hesitancy may be influenced by several factors, including complacency, convenience, and confidence. Complacency occurs when the perceived risks of vaccine-preventable diseases are low and vaccination is not considered a necessary or important preventive measure. Convenience refers to factors that influence vaccination decisions based on the accessibility and availability of vaccines, including physical availability and geographic access. Confidence encompasses trust in the effectiveness and safety of vaccines^7^ ^,^ ^8^.

To address the emerging challenges associated with declining VC and to develop innovative strategies, it is essential to consider the continuous evolution of disease epidemiological profiles, the rapid advancement of scientific knowledge, and the complex dynamics of contemporary society^9^. Surveillance of vaccine-preventable diseases requires rigorous and systematic assessments, as well as the development of strategic recommendations for health managers, to support the implementation of corrective actions based on the prioritization of municipalities according to their epidemiological risk classification^10^.

The identification of priority areas for the implementation of strategies aimed at improving immunization indicators can be achieved through the use of risk classification methodologies^10^. To address the challenges associated with low VC, it is essential to conduct a comprehensive situational analysis at the local level, as well as to identify pockets of susceptible individuals and areas most vulnerable to the transmission of vaccine-preventable diseases^2^ ^,^ ^10^.

Given the critical importance of VC to public health, it is relevant to examine their epidemiological situation in the municipality of São Paulo, the most populous city in the country, due to its substantial influence on the national health landscape.

Identifying regions with greater vulnerability to the transmission of vaccine-preventable diseases using rigorous criteria beyond VC may represent a complementary approach for establishing priorities and guiding interventions aimed at improving VC. In this context, the objective of the present study was to analyze the risk classification for the transmission of vaccine-preventable diseases among children under 2 years of age in the municipality of São Paulo, Brazil.

## METHODS

This ecological epidemiological study was conducted using secondary VC data for nine immunobiologicals included in the recommended immunization schedule for children under 2 years of age in the municipality of São Paulo, Brazil, between 2022 and 2023. The measles, mumps, and rubella (MMR) vaccine was analyzed separately for the first (D1) and second (D2) doses.

The municipality of São Paulo has a resident population of approximately 11.45 million inhabitants and the highest population density in Brazil, with 7,528.26 inhabitants per km^2^ across a territorial area of 1,521.202 km^2^.^11^ The municipality is administratively organized into 28 Health Surveillance Units (*Unidades de Vigilância em Saúde* - UVIS) and their respective Regional Health Coordinating Offices (*Coordenadorias Regionais de Saúde* - CRS). UVIS are responsible for environmental, sanitary, and epidemiological surveillance activities^12^. Primary Health Care Units (*Unidades Básicas de Saúde* - UBS) are distributed across the CRS and UVIS, totaling 470 facilities^13^.

The immunobiologicals selected for this study were as follows: oral rotavirus vaccine (considering both the monovalent rotavirus vaccine offered by the Brazilian Unified Health System (*Sistema Único de Saúde* - SUS) and the pentavalent rotavirus vaccine available through the private sector), meningococcal C vaccine (including the second dose of the meningococcal ACWY vaccine), pneumococcal vaccine (including the second dose of the 10-valent pneumococcal vaccine offered by SUS and the second dose of the 13-valent pneumococcal vaccine administered in the private sector), pentavalent vaccine (considering the third dose of the pentavalent vaccine used in SUS or, equivalently, the third dose of the hexavalent vaccine administered in the private sector), poliovirus vaccine (considering the third dose of the Inactivated Poliovirus Vaccine (IPV) or Oral Poliovirus Vaccine (OPV), as well as combination schedules containing inactivated poliovirus through hexavalent vaccines used in the private sector), first dose of the MMR vaccine (also considering the first dose of the measles, mumps, rubella, and varicella (MMRV) vaccine and the tetraviral vaccine), yellow fever vaccine (including single-dose, initial-dose, or first-dose schedules), second dose of the MMR vaccine (also considering the second dose of the MMRV vaccine and the second dose or single dose of the tetraviral vaccine), first dose of the hepatitis A vaccine, and first dose of the varicella vaccine (also considering the first dose or single dose of the tetraviral vaccine).

The BCG and hepatitis B vaccines were not included in this analysis because, in the municipality of São Paulo, they are routinely administered in hospital settings before newborn discharge. This differs from the administration of the other vaccines included in the childhood immunization schedule, which require access to primary health care services and may therefore reflect a different pattern of healthcare access. Consequently, the inclusion of these vaccines could influence the analyses and potentially introduce bias into the interpretation of VC data, thereby compromising comparability with the other immunobiologicals evaluated in this study.

VC data for 2022 were obtained from the TabNet platform of the Department of Informatics of the Brazilian Unified Health System (*Departamento de Informática do Sistema Único de Saúde* - DataSUS), website http://tabnet.datasus.gov.br. Data for 2023 were obtained from the Vaccination Coverage Panel for vaccines included in the National Immunization Schedule, on the website https://infoms.saude.gov.br/extensions/SEIDIGI_DEMAS_VACINACAO_CALENDARIO_NACIONAL_COBERTURA_RESIDENCIA/SEIDIGI_DEMAS_VACINACAO_CALENDARIO_NACIONAL_COBERTURA_RESIDENCIA.html. Both datasets were extracted on October 10, 2024, and were analyzed according to the municipality of residence.

Subsequently, vaccination coverage rates were classified according to the targets established by PNI and UVIS. Coverage targets were defined as greater than or equal to 90% for the human rotavirus vaccine and greater than or equal to 95% for the remaining immunobiologicals. Based on these thresholds, vaccination coverage was categorized as follows (Chart 1): very low (0 to less than 50%), low (greater than or equal to 50% and below the target), and adequate (greater than or equal to the target and less than 120%).

**Chart 1.** Summary of the parameters used to calculate the immunization indicators in this study.

Risk indicator for the transmission of vaccine-preventable diseases	Low risk: when vaccination coverage homogeneity for vaccines in the routine immunization schedule of children aged ≤1 year is 100%. Moderate risk: when vaccination coverage homogeneity for vaccines in the routine immunization schedule of children aged ≤1 year “ranges” between ≥75 and <100%. High risk: when vaccination coverage homogeneity for vaccines in the routine immunization schedule of children aged ≤1 year is <75%.
Braz et al.^10^ *	Very low: municipality with vaccination coverage homogeneity of 100% among children under 2 years of age. Low: municipality with vaccination coverage homogeneity ≥70% and <100% among children under 2 years of age and adequate vaccination coverage for the poliomyelitis, MMR, and pentavalent vaccines. Moderate: municipality with vaccination coverage homogeneity ≥70% and <100% among children under 2 years of age and VC below the target for up to 2 vaccines (poliomyelitis, MMR, or pentavalent). High: municipality with vaccination coverage homogeneity <70% among children under 2 years of age (regardless of VC). Very high: municipality with vaccination coverage homogeneity <70% among children under 2 years of age, a high dropout rate (>10%) for any of the 10 immunobiologicals^†^ evaluated, and a large population size and/or municipalities without vaccination records (for any vaccine), regardless of population size.

*Adaptation: Vaccination Coverage Homogeneity ≥70%; ^†^Nine immunobiologicals were analyzed, totaling 10 vaccination coverage indicators because the MMR vaccine was evaluated separately for the first dose (D1) and second dose (D2).

VC: Vaccination Coverage.

Source: Prepared by the authors.

The municipality, stratified by UVIS, was classified according to the Risk Indicator for the Transmission of Vaccine-Preventable Diseases (*Indicador de Risco de Transmissão de Doenças Imunopreveníveis* - RTDI), as described in the Health Surveillance Guide^14^. In addition, a classification of the risk of transmission of vaccine-preventable diseases was applied based on the methodology proposed by Braz^10^ and adapted for the purposes of this study.

The data were analyzed using the R statistical software package. To assess whether there was a significant reduction in the risk classifications for the reintroduction of vaccine-preventable diseases in the municipality of São Paulo between 2022 and 2023, according to UVIS, the paired Wilcoxon signed-rank test was applied. A significance level of 5% was adopted for all statistical analyses.

This study used publicly available data with no individual-level identifiers; therefore, approval by a Research Ethics Committee was not required.

## Data availability statement:

The complete dataset supporting the findings of this study is available from the corresponding author upon reasonable request. The dataset is not publicly available because it was generated through the integration of multiple databases performed by the authors.

## RESULTS

**Figure 1.**
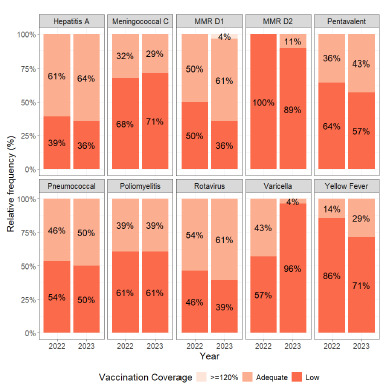
Percentage of vaccination coverage in the municipality of São Paulo, São Paulo (SP), Brazil, 2022 and 2023.

Source: Prepared by the authors.

Regarding the classification of VC rates across the 28 UVIS in the municipality of São Paulo, all UVIS (100%) were classified as having low VC (≥50% and below the target) for the second dose of the MMR vaccine in 2022. In the same year, the hepatitis A and human rotavirus vaccines achieved adequate VC in 61 and 54% of the UVIS, respectively. In 2023, an overall improvement in VC was observed across the UVIS; however, the meningococcal C and varicella vaccines showed a decline in the number of UVIS that achieved adequate VC rates (Figure 1).

**Figure 2.**
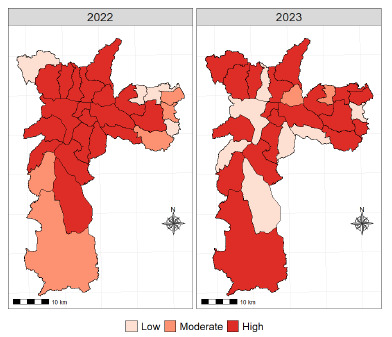
Risk indicator for the transmission of vaccine-preventable diseases according to year and Health Surveillance Units. São Paulo (SP), Brazil.

Source: Prepared by the authors.

Regarding the risk classification for the reintroduction of vaccine-preventable diseases, the proportion of UVIS classified as having a “low” risk according to the RTDI increased from 14.3% in 2022 to 25% in 2023. Conversely, the proportion classified as “medium” risk decreased from 17.9% in 2022 to 7.1% in 2023. The proportion of UVIS classified as “high” risk remained unchanged at 67.9% in both years. No statistically significant difference in RTDI classification was observed between 2022 and 2023 (Table 1 and Figure 2).

**Table 1.** Risk classification for the reintroduction of vaccine-preventable diseases in the Health Surveillance Units of the municipality of São Paulo, according to year and UVIS, São Paulo, 2022 and 2023.

	Years		p-value
	2022	2023	
	n (%)	n (%)	
RTDI Risk Classification^14^ for children ≤1 year of age			
Low	4 (14.3)	7 (25)	0.077
Moderate	5 (17.9)	2 (7.1)	
High	19 (67.9)	19 (67.9)	
BRAZ Risk Classification^10^ for children <2 years of age			
Very low and low	0 (0)	3 (10.7)	0.211
Moderate	10 (35.7)	6 (21.4)	
High and very high	18 (64.3)	19 (67.9)	

Source: Prepared by the authors.

**Figure 3.**
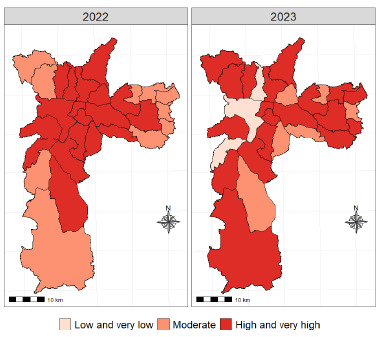
Classification of the risk of transmission of vaccine-preventable diseases, according to year and Health Surveillance Units, as described by Braz, São Paulo (SP), Brazil.

Source: Prepared by the authors.

Regarding the risk classification for the reintroduction of vaccine-preventable diseases among children under 2 years of age, based on the methodology proposed by Braz and stratified by UVIS, an increase was observed in the proportion of UVIS classified as having a “low and very low” risk of transmission, rising from 0% in 2022 to 10.7% in 2023. The proportion of UVIS classified as having a “medium” risk decreased from 35.7 to 21.4%. However, the proportion classified as having a “high and very high” risk increased from 64.3% in 2022 to 67.9% in 2023 (Table 1 and Figure 3).

## DISCUSSION

Analysis of VC indicators showed that the hepatitis A vaccine had the highest proportion of adequate coverage among the UVIS in the municipality of São Paulo during the study period. In 2022, the rotavirus vaccine was the immunobiological with the second-highest proportion of UVIS achieving adequate coverage. In 2023, the rotavirus vaccine continued to demonstrate a high proportion of adequate coverage, and the D1 of the MMR vaccine also achieved one of the highest percentages of adequate coverage among the UVIS.

In 2022, it was observed that all UVIS presented low coverage for the MMR vaccine D2; however, in 2023, the varicella vaccine showed the highest percentage of low coverage. Comparing 2022 and 2023 with respect to the risk classification for the reintroduction of vaccine-preventable diseases, a reduction in risk was observed, with an increase in the proportion of UVIS classified as “low” risk according to RTDI. However, an increase was observed in the “high” and “high and very high” risk classifications according to the Braz classification.

The scenario of VC rates below target levels observed in the municipality of São Paulo is also a reality in other regions of Brazil^15^. A study conducted in Minas Gerais demonstrated a decreasing trend in the coverage rates of at least five evaluated immunobiologicals, while the pentavalent vaccine showed a declining trend in vaccination coverage across a large portion of the state^16^. In the present study, a reduction in the proportion of UVIS with low VC for the pentavalent vaccine was observed when comparing 2022 and 2023, demonstrating an improvement in vaccination activities within the territories covered by the UVIS.

The hepatitis A vaccine showed adequate VC in most of the UVIS in the municipality of São Paulo, a finding that may be explained by efforts to provide vaccination to children throughout the country. In 2014, the MoH, through PNI, implemented hepatitis A vaccination, offering a single dose of the monovalent inactivated virus vaccine to children aged 15 to 24 months^17^. In 2017, vaccination was expanded to include children under 5 years of age in order to protect those who had not been vaccinated at the beginning of the program in 2014^18^. Furthermore, an increase in hepatitis A VC was also reported at the national level^19^.

Rotavirus VC increased among the UVIS in 2023 compared with 2022. Globally, rotavirus, the leading etiological agent of acute gastroenteritis (AGE), is estimated to cause 125 million cases annually, resulting in approximately 2 million hospitalizations and 600,000 deaths^20^. Studies have demonstrated reductions in morbidity and mortality associated with AGE following the introduction of the rotavirus vaccine. In São Paulo, a 59% reduction in hospitalizations due to rotavirus-related AGE was observed during the post-vaccination period, that is, after the introduction of the vaccine^21^.

The second dose (D2) of the MMR vaccine was the immunobiological that showed the lowest vaccination coverage in all UVIS in the municipality in 2022 and in 89% of the UVIS in 2023. An analysis aimed at identifying areas with declining VC for BCG, poliomyelitis, and MMR vaccines in Brazil found that 2013 had the lowest MMR vaccination coverage, with 77.1% of children up to one year of age vaccinated nationwide. Furthermore, the greatest annual reduction in the number of vaccinated children was observed, reaching 2.7% per year^15^. In efforts to understand the decline in VC in Brazil between 2010 and 2020, it is important to note that the previously reported substantial reduction in MMR vaccination coverage increased population vulnerability, contributing to the reemergence of measles in 2018. This event may also have been associated with the migration of Venezuelan populations into the country^22^. Cases were reported, based on partial data from 2018, in the states of Roraima, Amazonas, Rio Grande do Sul, São Paulo, Rondônia, and Rio de Janeiro^22^.

The decline in VC rates, together with the resurgence of vaccine-preventable diseases, requires in-depth discussion, as identifying the factors that have caused and contributed to this situation in a country such as Brazil is essential^23^. Furthermore, knowledge about vaccination (access to relevant information and the ability to understand such information) and confidence (in vaccination, whether in vaccines in general or in one’s own ability to make decisions regarding vaccine uptake), are mechanisms frequently cited in discussions of factors influencing vaccine acceptance^24^.

Furthermore, the role of vaccine hesitancy in the decline of VC is noteworthy. The dissemination of misinformation contributes to the spread of inaccurate information, often emphasizing potential adverse effects allegedly associated with vaccination^23^. In addition, reports of vaccine side effects and scientifically unsubstantiated claims regarding hypothetical health conditions attributed to vaccination may increase skepticism about vaccine effectiveness^25^. A review identified contextual influences, individual and group influences, and vaccine-specific issues among the factors associated with vaccine hesitancy^26^.

In this context, although Brazil has been recognized internationally for its PNI since 1973, the country achieved the recommended VC target only in 2015. The dissemination of misinformation and sensationalized, scientifically unsupported claims through social media has contributed to fear and distrust regarding vaccination, resulting in the reemergence of diseases such as measles and yellow fever^22^. In addition, logistical complexity, occasional shortages of certain vaccines, and limited operating hours at vaccination centers are factors that may contribute to the low VC observed in recent years^27^ ^,^ ^28^.

When analyzing the temporal and spatial distribution of poliomyelitis VC in Brazilian states, spatial clusters of municipalities with high vaccination coverage rates were identified in the state of São Paulo between 2014 and 2021^29^. In 2023, the risk classification for poliomyelitis reintroduction in Brazil changed from “very high” to “high.” In this context, vaccination coverage in 2023 showed an increasing trend compared with 2020, 2021, and 2022. Furthermore, immunobiologicals administered to children at 1 year of age exhibited a significant increase in coverage compared with the respective previous years^29^.

The MoH has reported an increase in VC in Brazil and in the municipality of São Paulo throughout 2023. Furthermore, it has continuously implemented strategies aimed at restoring high vaccination coverage and reducing pockets of unvaccinated individuals within its territory^30^ ^,^ ^31^. In 2023, financial resources were also allocated to support the development of actions designed to reduce the population’s susceptibility to the reintroduction of diseases previously eliminated through vaccination efforts in the national territory and to reinforce the commitment to recovering VC^31^ ^,^ ^32^.

The implementation of multivaccination campaigns in a regionalized manner across each federative unit and its respective municipalities contributes to increasing VC and reducing the risk of the (re)introduction or dissemination of vaccine-preventable diseases in Brazil^32^.

Similarly, the Municipal Immunization Program of São Paulo (*Programa Municipal de Imunizações* - PMI/SP) has continuously implemented various strategies to recover VC, particularly following the impacts of the COVID-19 pandemic. In 2022, in the municipality of São Paulo, intensive vaccination campaigns and awareness-raising initiatives^33^ contributed to significant improvements in VC for poliomyelitis and hepatitis A. Thus, increased adherence to vaccination contributed to reducing the risk of outbreaks and protecting the population against vaccine-preventable diseases^34^.

Finally, the main limitation of this study is the use of secondary data. However, despite this potential limitation, it was possible to conduct a robust and comprehensive analysis of VC in the municipality of São Paulo, using rigorous methods to ensure the internal validity of the results.

The findings presented may contribute to a better understanding of the VC scenario, providing important insights for public health interventions related to immunization and the risk classification of the reintroduction of vaccine-preventable diseases.

For most of the vaccines analyzed, an improvement in the VC scenario was observed among the UVIS in 2023. However, variations in VC were identified according to the vaccine and the year analyzed, particularly for the MMR vaccine, which did not achieve adequate coverage in any region of the municipality in 2022. These findings reinforce the need for continued regional actions to achieve the recommended targets, given the indications of recovery in vaccination coverage observed in both the municipality and the country.
