# Supplementary material for: Classification of the risk of transmission of vaccine-preventable diseases among children under 2 years of age in the municipality of São Paulo, Brazil
Source: Rev Bras Epidemiol. 2026 Aug 7;29:e260035. doi: 10.1590/1980-549720260035 (PMC13449748; doi:10.1590/1980-549720260035)
Supplement: Translation

# Translation: Classificação do risco de transmissão de doenças preveníveis por vacinação para crianças menores de 2 anos no município de São Paulo, Brasil

**DOI:** 10.1590/1980-549720260035.2

**Authors:** Mariana de Souza Araújo, José Elisomar Silva de Santana, Melissa Palmieri, Luciana Ursini Nunes, Thaís Moreira Oliveira, Maria Eduarda Viana Oliveira, Guilherme Augusto Veloso, Thales Philipe Rodrigues da Silva, Fernanda Penido Matozinhos

**Affiliations:** I Prefeitura Municipal de São Paulo, Secretaria Municipal da Saúde, Coordenadoria de Vigilância em Saúde - São Paulo (SP), Brasil.; II Prefeitura Municipal de São Paulo, Secretaria Municipal da Saúde, Coordenadoria de Vigilância em Saúde, Programa Municipal de Imunizações - São Paulo (SP), Brasil.; III Universidade Federal de Minas Gerais, Escola de Enfermagem - Belo Horizonte (MG), Brasil.; IV Universidade Federal Fluminense, Instituto de Matemática e Estatística, Departamento de Estatística - Niterói (RJ), Brasil.

**Keywords:** Cobertura vacinal, Gestão de risco, Vacinação, Vigilância em saúde pública

## Abstract

**Objetivo:**

Analisar a classificação de risco de transmissão de doenças preveníveis por ação de vacinação para crianças menores de 2 anos em município de grande porte brasileiro nos anos de 2022 e 2023.

**Métodos:**

Estudo epidemiológico, ecológico, com dados secundários de Cobertura Vacinal (CV) de nove imunobiológicos recomendados para o calendário vacinal de crianças menores de 2 anos. As doses de imunobiológico foram acessadas pelo Sistema de Informações do Programa Nacional de Imunizações (SI-PNI); já os dados foram coletados do site do Ministério da Saúde, filtrados por residência. Os dados foram analisados pelo pacote R.

**Resultados:**

No ano de 2022, 100% das Unidades de Vigilância em Saúde (UVIS) apresentaram CV baixa para a vacina tríplice viral D2. No mesmo ano, para as vacinas hepatite A e rotavírus humano, 61% e 54% das UVIS apresentaram CV adequada, respectivamente. No ano de 2023, observou-se melhora das CV, exceto para a vacina meningococo C e varicela, que apresentaram redução no número de UVIS que alcançaram CV adequada. Para a classificação de risco para reintrodução de doenças imunopreveníveis, nos anos de 2022 e 2023, verificou-se um aumento na porcentagem da classificação “baixo e muito baixo”, de 0% em 2022 para 10,7% em 2023.

**Conclusão:**

Para a maioria das vacinas analisadas houve melhora no cenário das CV nas UVIS em 2023. Todavia, há variações nas CV por vacina e por ano analisado. Reforça-se a necessidade de continuidade das ações regionais para o alcance das metas desejadas, devido a indícios da reconquista das coberturas vacinais.

## INTRODUÇÃO

Criado em 1973, o Programa Nacional de Imunizações (PNI) do Brasil é, no âmbito da saúde, uma das maiores conquistas de saúde pública^1^. Oferece vacinas com qualidade para a população a nível nacional e contribui com a melhoria da saúde pública no país^2^.

Apesar do sucesso inegável do PNI ao longo dos anos, a partir de 2016, observou-se uma queda progressiva e preocupante das coberturas vacinais no mundo e no Brasil^1^, que foi potencializada pela pandemia da covid-19^3^. Segundo dados do Ministério da Saúde (MS), as taxas de Cobertura Vacinal (CV), que chegaram a 97% em 2015, caíram para 75% em 2020^4^. Em 2021, de acordo com a Organização Mundial da Saúde (OMS), no Brasil, na população infantil, quase 26% desse público não recebeu nenhuma dose de vacina^4^.

O declínio das CV é resultado de múltiplos fatores^1^, entre eles a hesitação vacinal^5^, definida como a resistência ou recusa em vacinar, apesar da disponibilidade de vacinas^6^. Em 2019, a OMS estabeleceu a “hesitação em relação à vacina” como uma das dez maiores ameaças à saúde global^6^. A hesitação pode ser influenciada por várias questões, como: a complacência, que se dá com a baixa compreensão dos riscos de doenças preveníveis por vacinas e quando a vacinação não é considerada uma conduta prudente e crucial; conveniência, significativa na decisão da vacinação e que varia conforme a praticidade e a facilidade no alcance da vacina, envolvendo fatores como disponibilidade física e acesso geográfico; e confiança, que inclui a convicção na efetividade e na segurança das vacinas^7^ ^,^ ^8^.

Para enfrentar os desafios emergentes diante de novas ameaças associadas a quedas das coberturas vacinais e também para o desenvolvimento de estratégias inovadoras, é preciso considerar a evolução contínua do perfil epidemiológico das doenças, o avanço acelerado do conhecimento científico e as características complexas da sociedade^9^. A vigilância das CV exige avaliações sistemáticas rigorosas e a emissão de recomendações estratégicas aos gestores, visando à implementação de ações corretivas com base na priorização dos municípios, conforme a classificação de risco epidemiológico^10^.

A identificação de áreas prioritárias para estabelecimento de estratégias que visem à melhoria dos indicadores de imunização pode ser realizada por meio da metodologia de classificação de risco^10^. Para superar os desafios das baixas CV, é essencial realizar uma análise situacional detalhada nas administrações locais, além de identificar bolsões de indivíduos suscetíveis e áreas mais vulneráveis à transmissão de doenças evitáveis por vacinação^2^ ^,^ ^10^.

Considerando as CV de suma importância para a manutenção da saúde coletiva, é relevante ressaltar a situação das CV do município de São Paulo, que possui a maior densidade demográfica do país, impactando o cenário nacional.

A determinação de regiões com maior vulnerabilidade à disseminação de doenças preveníveis por vacinação, avaliada por critérios rigorosos, para além da CV, pode constituir-se em abordagem complementar para estabelecer prioridades e direcionar intervenções para a reconquista das CV. Diante do exposto, o objetivo deste estudo foi analisar a classificação de risco de transmissão de doenças preveníveis por ação de vacinação para crianças menores de 2 anos no município de São Paulo, Brasil.

## MÉTODOS

Estudo epidemiológico, ecológico, realizado com dados secundários de CV de nove imunobiológicos recomendados para o calendário vacinal de crianças menores de 2 anos no município de São Paulo, Brasil, entre 2022 e 2023. A vacina tríplice viral foi analisada considerando separadamente a primeira (D1) e segunda doses (D2).

O município de São Paulo possui uma população residente de aproximadamente 11,45 milhões de pessoas e a maior densidade demográfica do país, com 7.528,26 habitantes por km^2^ e uma área territorial de 1.521,202 km^2^.^11^ O município é organizado por 28 Unidades de Vigilância em Saúde (UVIS) e suas respectivas Coordenadorias Regionais de Saúde (CRS). As UVIS atuam nas áreas de vigilância ambiental, vigilância sanitária e vigilância epidemiológica^12^. As Unidades Básicas de Saúde (UBS) são distribuídas por CRS e por UVIS, totalizando 470^13^.

Os imunobiológicos selecionados neste estudo foram: vacina oral contra o rotavírus (considerou-se tanto a vacina rotavírus monovalente ofertada pelo Sistema Único de Saúde (SUS) - quanto a vacina rotavírus pentavalente ofertada pela rede privada), vacina contra a doença meningocócica C (considerou-se, também, a segunda dose da meningocócica ACWY), vacina contra a doença pneumocócica (considerou-se a segunda dose da vacina pneumocócica 10-valente ofertada pelo SUS e a segunda dose da pneumocócica 13-valente utilizada na rede privada), vacina pentavalente (considerou-se a terceira dose da vacina pentavalente utilizada no SUS ou, de forma equivalente, a terceira dose da vacina hexavalente utilizada na rede privada), vacina contra a poliomielite (considerou-se a terceira dose da Vacina Inativada Poliomielite (VIP) ou da Vacina Oral Poliomielite (VOP), bem como esquemas combinados contendo poliovírus inativado presentes nas vacinas hexavalentes utilizadas na rede privada), primeira dose da vacina tríplice viral (considerou-se, também, a primeira dose das vacinas quádrupla viral e tetra viral), vacina contra a febre amarela (considerou-se aquelas de dose única, inicial ou primeira dose), segunda dose da vacina tríplice viral (considerou-se, também, a segunda dose da quádrupla viral e segunda dose ou dose única da tetra viral), primeira dose da vacina contra a hepatite A e primeira dose da vacina contra a varicela (considerou-se, também, a primeira dose ou dose única da vacina tetra viral).

As vacinas BCG e hepatite B não foram incluídas na presente análise, pois em São Paulo a sua administração ocorre rotineiramente no ambiente hospitalar, antes da alta do recém-nascido. Essa característica difere das demais vacinas do calendário infantil, que dependem da procura pelos serviços de atenção primária, e tal situação pode introduzir um padrão diferente de acesso. Assim, sua inclusão poderia causar alterações nas análises e possivelmente gerar vieses na interpretação dos resultados relacionados à CV, de forma a comprometer a comparabilidade com as demais vacinas analisadas no estudo.

As CV foram extraídas, no ano de 2022, via Tabnet/Departamento de Informação e Informática do SUS (DataSUS), site http://tabnet.datasus.gov.br; e para o ano de 2023, via Painel da Cobertura Vacinal das vacinas presentes no Calendário Nacional, no site https://infoms.saude.gov.br/extensions/SEIDIGI_DEMAS_VACINACAO_CALENDARIO_NACIONAL_COBERTURA_RESIDENCIA/SEIDIGI_DEMAS_VACINACAO_CALENDARIO_NACIONAL_COBERTURA_RESIDENCIA.html. Ambas as extrações foram realizadas no dia 10 de outubro de 2024, por residência.

Posteriormente, as CV foram organizadas segundo as metas preconizadas pelo PNI e pelas UVIS, como maior ou igual a 90% para a vacina contra o rotavírus humano e maior ou igual a 95% para os demais imunobiológicos, obtendo-se a seguinte categorização (Quadro 1): muito baixa (0 a menor que 50%), baixa (maior ou igual a 50% e menor que a meta) e adequada (maior ou igual a meta a menor que 120%).

**Quadro 1.** Síntese dos parâmetros utilizados para cálculo dos indicadores de imunização neste estudo.

Indicador de risco de transmissão de doenças imunopreveníveis	Baixo risco: quando a homogeneidade de coberturas das vacinas do calendário das crianças com idade menor ou igual a 1 ano for=100%. Médio risco: quando a homogeneidade de coberturas das vacinas do calendário das crianças com idade menor ou igual a 1 ano “flutuar” entre ≥75 e <100%. Alto risco: quando a homogeneidade de coberturas das vacinas do calendário das crianças com idade menor ou igual a 1 ano for <75%.
Braz et al.^10^ *	Muito baixo: município com HCV em menores de 2 anos=100%. Baixo: município com HCV maior ou igual a 70% e menor que 100% em menores de 2 anos, com cobertura vacinal adequada para as vacinas contra poliomielite, tríplice viral e pentavalente. Médio: município com HCV maior ou igual a 70% e menor que 100% em menores de 2 anos, com CV abaixo da meta para até 2 vacinas (poliomielite, tríplice viral ou pentavalente). Alto: município com HCV menor que 70% em menores de 2 anos (independentemente da CV). Muito alto: município com HCV menor que 70% em menores de 2 anos, taxa de abandono alta (maior que 10%) para qualquer um dos 10 imunobiológicos^†^ avaliados e com grande porte populacional e/ou municípios sem registro vacinal (qualquer vacina), independentemente do porte populacional.

*Adaptação: Homogeneidade de Cobertura Vacinal maior ou igual a 70%; ^†^Foram analisados nove imunobiológicos, totalizando 10 coberturas vacinais, uma vez que a vacina tríplice viral foi avaliada separadamente para a primeira (D1) e segunda dose (D2).

HCV: Homogeneidade de Cobertura Vacinal; CV: Cobertura Vacinal.

Fonte: Elaborado pelos autores.

O município, estratificado pelas UVIS, recebeu uma classificação quanto ao Indicador de Risco de Transmissão de Doenças Imunopreveníveis (RTDI), disponível no Guia de Vigilância em Saúde^14^, e segundo classificação quanto ao risco de transmissão de doenças preveníveis por ação de vacinação, como descrito em Braz ^10^ e adaptado.

Os dados foram analisados por meio do pacote R. Para verificar se houve redução significativa nas classificações de risco para reintrodução de doenças preveníveis por ação de vacinação no município de São Paulo entre os anos de 2022 e 2023 segundo as UVIS, utilizou-se o teste de postos sinalizados de Wilcoxon pareado. Adotou-se o nível de significância de 5% para o procedimento analítico, em sua totalidade.

Para realização deste estudo, foram utilizados dados de domínio público e sem identificação individual, dispensando-se, assim, a aprovação de um Comitê de Ética e Pesquisa.

## Declaração de disponibilidade de dados:

Todo o conjunto de dados que dá suporte aos resultados deste estudo está disponível mediante solicitação ao autor correspondente. O conjunto de dados não está publicamente disponível devido à integração de bancos de dados realizados pelos autores.

## RESULTADOS

Em relação à classificação das CV segundo as 28 UVIS do município de São Paulo, observou-se que, no ano de 2022, 100% das UVIS apresentaram CV classificada como baixa (maior ou igual a 50% e menor que a meta) para a vacina tríplice viral D2. Em relação à classificação das CV nas 28 UVIS do município de São Paulo, observou-se que, em 2022, 100% das UVIS apresentaram CV baixa (maior ou igual a 50% e inferior à meta) para a vacina tríplice viral D2. Ainda nesse ano, as vacinas hepatite A e rotavírus humano apresentaram CV adequada em 61 e 54% das UVIS, respectivamente. Em 2023, observou-se melhora no cenário das CV nas UVIS, exceto para as vacinas meningocócica C e varicela, que apresentaram redução no número de UVIS que alcançaram CV adequada (Figura 1).

**Figura 1.** Porcentagem de cobertura vacinal do município de São Paulo, São Paulo (SP), Brasil, 2022 e 2023.

Fonte: Elaborado pelos autores.

Em relação à classificação de risco para reintrodução de doenças preveníveis por ação de vacinação, observou-se que a proporção de UVIS classificadas como “baixo” na classificação RTDI aumentou de 14,3% em 2022 para 25% em 2023. A proporção de UVIS classificadas como “médio” segundo a RTDI diminuiu de 17,9% em 2022 para 7,1% em 2023. A categoria “alto” não apresentou alterações na porcentagem das UVIS classificadas, permanecendo 67,9% em 2022 e 2023, sem diferença estatisticamente significativa (Tabela 1 e Figura 2).

**Tabela 1.** Classificação de risco para a reintrodução de doenças preveníveis por vacinas das Unidades de Vigilância em Saúde do município de São Paulo, segundo ano e UVIS, São Paulo, 2022 e 2023.

	Anos		p-valor
	2022	2023	
	n (%)	n (%)	
Classificação de Risco (RTDI)^14^ para ≤1 ano			
Baixo	4 (14,3)	7 (25)	0,077
Médio	5 (17,9)	2 (7,1)	
Alto	19 (67,9)	19 (67,9)	
Classificação de Risco (BRAZ)^10^ para menores de 2 anos			
Muito baixo e baixo	0 (0)	3 (10,7)	0,211
Médio	10 (35,7)	6 (21,4)	
Alto e muito alto	18 (64,3)	19 (67,9)	

Fonte: Elaborado pelos autores.

**Figura 2.** Indicador de risco de transmissão de doenças preveníveis por vacinas segundo ano e Unidades de Vigilância em Saúde. São Paulo (SP), Brasil.

Fonte: Elaborado pelos autores.

Para a classificação de risco para reintrodução de doenças preveníveis com vacinação de acordo com o método Braz para menores de 2 anos segundo as UVIS, nos anos de 2022 e 2023, observou-se um aumento na porcentagem de UVIS classificadas como “baixo e muito baixo” para transmissão de doenças preveníveis por ação de vacinação, de 0% em 2022 para 10,7% em 2023. A proporção das UVIS classificadas como “médio” apresentou redução de 35,7 para 21,4%. Entretanto, entre as UVIS classificadas como “alto e muito alto”, observou-se aumento, de 64,3% em 2022 para 67,9% em 2023 (Tabela 1 e Figura 3).

**Figura 3.** Classificação quanto ao risco de transmissão de doenças preveníveis por vacinas, segundo ano e Unidades de Vigilância em Saúde, como descrito em Braz, São Paulo (SP), Brasil.

Fonte: Elaborado pelos autores.

## DISCUSSÃO

As análises dos indicadores de CV evidenciaram que a vacina contra hepatite A foi a que apresentou a maior porcentagem de cobertura adequada nas UVIS para os anos analisados na cidade de São Paulo. Em 2022, o segundo imunobiológico com maior proporção de CV adequada entre as UVIS foi contra o rotavírus e, em 2023, além deste, a tríplice viral D1 obteve maior porcentagem de CV adequada entre as UVIS.

Em 2022, observou-se que todas as UVIS apresentaram CV baixa para a vacina tríplice viral D2; já no ano de 2023, o imunobiológico contra a varicela alcançou a maior porcentagem de baixa cobertura. Comparando-se os anos 2022 e 2023 acerca da classificação de risco para reintrodução de doenças preveníveis por ação de vacinação, houve uma redução no risco, com um aumento na proporção de UVIS em risco “baixo” pela classificação RTDI. Todavia, houve aumento da classificação “alto” e “alto e muito alto” no risco pela classificação de Braz.

O cenário de CV aquém das metas encontrado no município de São Paulo também é uma realidade em outros locais brasileiros^15^. Estudo realizado em Minas Gerais demonstrou tendência decrescente na CV de pelo menos cinco imunobiológicos avaliados, enquanto a vacina pentavalente apresentou tendência decrescente de CV em grande parte do território^16^. No presente estudo, houve diminuição da CV baixa para a vacina pentavalente entre as UVIS quando comparados os anos de 2022 e 2023, o que demonstra melhora nas ações de vacinação no território das UVIS.

A vacina contra a hepatite A apresentou CV adequada na maior parte das UVIS do município de São Paulo, e tal fato pode ser justificado pelos esforços do país em ofertar a vacinação para as crianças em todo o território. Em 2014, o MS, por meio do PNI, implantou o programa de vacinação contra hepatite A, que disponibilizava dose única da vacina monovalente de vírus inativado para crianças entre 15 e 24 meses de vida^17^. Em 2017, ocorreu a ampliação da vacinação para crianças com menos de 5 anos de idade, para proteção das crianças que não foram vacinadas no início do programa em 2014^18^. Ademais, o aumento da CV para a vacina contra hepatite A também foi relatado em âmbito nacional^19^.

A CV contra o rotavírus aumentou nas UVIS em 2023 em relação a 2022. No cenário mundial, estima-se que, anualmente, ocorram 125 milhões de casos pelo rotavírus, principal agente etiológico causador de Gastroenterite Aguda (GEA), resultando em cerca de 2 milhões de internações e 600 mil óbitos^20^. Autores comprovam a redução da morbimortalidade associada a GEA após introdução da vacina contra o rotavírus. Em São Paulo, observou-se diminuição de 59% no número de hospitalizações por GEA por rotavírus no período pós-vacina, ou seja, após a introdução da vacina^21^.

A segunda dose da vacina tríplice viral, que fornece imunização contra caxumba, sarampo e rubéola, foi a que obteve em todas as UVIS do município CV baixa no ano de 2022, e em 89% no ano de 2023. Uma análise para evidenciar áreas com queda da CV de BCG, poliomielite e tríplice viral no Brasil apontou que 2013 foi o ano com menor cobertura para a tríplice viral, em que 77,1% das crianças com até um ano de idade foram vacinadas no país. Além disso, registrou-se, no decorrer dos anos, o maior valor de redução no número de vacinados, alcançando 2,7% a cada ano^15^. Em busca da compreensão da queda de CV no Brasil entre os anos de 2010 e 2020, destaca-se que, devido à notável queda da CV da tríplice viral relatada anteriormente, houve aumento da vulnerabilidade, viabilizando o retorno do sarampo em 2018 - isso pode estar associado também ao processo de imigração de venezuelanos no país^22^. Houve registros de casos, com dados parciais de 2018, nos estados de Roraima, Amazonas, Rio Grande do Sul, São Paulo, Rondônia e Rio de Janeiro^22^.

As quedas de CV associadas ao ressurgimento de casos de doenças preveníveis por ação de vacinação necessitam de profunda discussão, pois é imprescindível identificar os fatores que as ocasionaram e contribuíram para que ocorressem em um país como o Brasil^23^. Ademais, o conhecimento sobre vacinação (acesso a informações relevantes e/ou capacidade de entender informações) e a confiança (na vacinação em geral, ou em si mesmo para tomar decisões sobre a adesão) são considerados mecanismos frequentemente citados para discutir fatores que influenciam a aceitação de vacinas^24^.

Destaca-se, ainda, o papel da hesitação vacinal na queda das CV. A disseminação de informações falsas fomenta a propagação de informações inverídicas, com o foco em possíveis riscos negativos que as vacinas podem submeter aos indivíduos^23^. Além disso, narrativas de efeitos colaterais das vacinas e explicações sem teor científico de lesões cutâneas hipotéticas advindas da vacinação aumentam o questionamento da eficácia das vacinas^25^. Revisão aponta que, entre os fatores associados à hesitação vacinal, têm-se influências contextuais, influências individuais e de grupo, e questões específicas da vacina^26^.

Nesse contexto, embora o Brasil seja referência mundial com o PNI desde 1973, o país só atingiu a meta de CV recomendada em 2015. A disseminação de notícias falsas (fake news) e argumentos sensacionalistas sem base científica nas redes sociais tem gerado pânico e desconfiança, resultando no reaparecimento de doenças como o sarampo e a febre amarela^22^. A complexidade logística, o desabastecimento pontual de algumas vacinas e as restrições de horários das salas de vacina também são fatores que podem contribuir para a baixa CV dos últimos anos^27^ ^,^ ^28^.

Ao se analisar a distribuição temporal e espacial da CV contra poliomielite nos estados brasileiros, no estado de São Paulo, identificaram-se aglomerados espaciais de municípios com altas taxas de cobertura vacinal contra poliomielite entre os anos de 2014 e 2021^29^. Em 2023, a classificação de risco de reintrodução de poliomielite no Brasil passou de “muito alto” para “alto” e, nesse contexto, nota-se tendência de aumento nas coberturas vacinais do ano de 2023 em relação aos anos de 2020, 2021 e 2022. Ademais, os imunobiológicos destinados à população de 1 ano de idade apresentaram aumento percentual significativo em relação aos respectivos anos^29^.

O MS relata o aumento das coberturas vacinais no Brasil e em São Paulo ao longo de 2023. Ainda, vem apresentando, continuamente, estratégias para a retomada de altas coberturas vacinais em seu território e redução dos bolsões de não vacinados^30^ ^,^ ^31^. Em 2023, também direcionou esforços financeiros para apoiar o desenvolvimento de ações para diminuir a suscetibilidade da população à reintrodução de doenças já eliminadas por meio de ação de vacinação no território nacional e reforçar o compromisso de recuperar as CV^31^ ^,^ ^32^.

A operacionalização das ações de multivacinação de forma regionalizada em cada unidade federada e respectivos municípios corrobora para a elevação das CV e contribui para redução do risco de (re)introdução ou disseminação de doenças preveníveis por vacinas no Brasil^32^.

De modo similar, o Programa Municipal de Imunizações (PMI/SP) implementa, continuamente, diversas estratégias para recuperar as CV, especialmente após os impactos da pandemia de covid-19. Em 2022, no município de São Paulo, por meio de campanhas intensivas e ações de conscientização^33^, a CV da poliomielite e hepatite A apresentou progressos significativos. Dessa forma, a ampliação da adesão à vacinação contribuiu para a redução do risco de surtos e para a proteção da população contra doenças preveníveis por vacinas^34^.

Por fim, o presente estudo apresenta como principal limitação a utilização de dados secundários. Todavia, apesar desta potencial limitação, foi possível uma análise robusta e abrangente das CV do município de São Paulo, com a utilização de técnicas rigorosas para garantia da validade interna dos resultados.

Os achados apresentados podem contribuir para o melhor entendimento do cenário de CV, com subsídios importantes para intervenções de saúde pública no âmbito da imunização e da classificação de risco de reintrodução de doenças preveníveis por vacinação.

Para a maioria das vacinas analisadas, em 2023 houve melhora no cenário das CV nas UVIS. Todavia, há variações nas CV por vacina e por ano analisado, com destaque para a vacina tríplice viral, que em 2022 não alcançou cobertura adequada em nenhuma das regiões do município. Reforça-se a necessidade de continuidade das ações regionais para o alcance das metas desejadas, uma vez que há indícios da reconquista das coberturas vacinais no município e país.
